# Encapsulation and Self-Superparasitism of *Pseudapanteles dignus* (Muesebeck) (Hymenoptera: Braconidae), a Parasitoid of *Tuta absoluta* (Meyrick) (Lepidoptera: Gelechiidae)

**DOI:** 10.1371/journal.pone.0163196

**Published:** 2016-10-12

**Authors:** María G. Luna, Nicolas Desneux, Marcela I. Schneider

**Affiliations:** 1 CEPAVE (CONICET-UNLP), La Plata, Argentina; 2 French National Institute for Agricultural Research (INRA), Sophia-Antipolis, France; Institute of Plant Physiology and Ecology Shanghai Institutes for Biological Sciences, CHINA

## Abstract

Endoparasitoids can be killed by host encapsulation, a cellular-mediated host immunological response against parasitism that involves hemocytes aggregation. As a counteracting strategy, many parasitoids can evade this host response through self-superparasitism. The objectives of this study were: 1) to describe the parasitoid *Pseudapanteles dignus* (Hymenoptera: Braconidae) early immature stages (egg and larva) encapsulation by the host *Tuta absoluta* (Lepidoptera: Gelechiidae), and 2) to determine the occurrence of self-superparasitism and the rate of escaping to encapsulation of this parasitoid. Knowledge of host-parasitoid immunological interaction is crucial when evaluating the potential of an endoparasitoid as a biological control candidate. Parasitoid-exposed *T*. *absoluta* larvae were dissected *in vivo* under light stereoscope microscope at 24-h intervals, for five days after exposition to detect encapsulation. The preimaginal stages of *P*. *dignus* and numbers of healthy and encapsulated immature parasitoids per host were recorded. Samples of parasitoid eggs and larvae were processed for SEM visualization of encapsulation. Necropsies evidenced that only the early first larval instar of *P*. *dignus* (up to 96 h-old) was partially or completely encapsulated. A non-melanized capsule, formed by layers of granulocyte-type hemocytes enveloping around the parasitoid body, was recorded. Approximately 50% of the parasitized *T*. *absoluta* larvae had significantly only one *P*. *dignus* egg, meanwhile supernumerary parasitization yielded up to seven immature parasitoids per host. The proportion of single-early first larval instar of *P*. *dignus* reached ≈ 0.5 and decreased significantly as the number of parasitoid individuals per host increased. *P*. *dignus* encapsulation and its ability to overcome with the host immune defense through self-superparasitism indicate that *T*. *absoluta* is a semi-permissive host for this parasitoid.

## Introduction

Parasitoid-host interactions involve biological and physiological processes. Regarding host defense against endoparasitoids, i.e. those which juvenile instars develop inside the host body, a cellular-mediated response is implicated in forming a multi-layered sheath of blood cells or hemocytes to its surface. Several differentiated types of hemocytes have been morphologically and functionally characterized, being the symmetrically-spread, adhesive, phagocytic granulocyte- type the most abundant blood cells in Lepidoptera. Granulocytes strongly adhere to the parasitoid’s surface and can form a capsule surrounding the parasitoid body totally, eliminating intruders by asphyxiation, starvation or physical prevention of development, or partially, what enables parasitoids to continue developing normally [1, 2]. Encapsulation can also produce inhibition of parasitoid embryonic development, extension of its developmental time, and reduction in the number of offspring [3, 4].

To avoid encapsulation, endoparasitoids display several mechanisms, such as superparasitism and injection of immune-suppressors (e.g. venoms and/or polydnaviruses) [5, 6]. Superparasitism may lead to overall reduced fitness; however, provigenic to moderate synovigenic not egg-limited solitary parasitoids can benefit from placing several eggs in a host, which leads to high levels of encapsulation and a consequent saturation of the host’s defense system, giving a chance for one individual to survive [7]. A balance of these processes determines the parasitism success and the host range of parasitoids [8].

Encapsulation may adversely affect biological control success, by preventing the effective establishment of exotic parasitoids in new regions, or by reducing the parasitoid´s efficacy against targeted pest(s) [9, 10]. A high incidence of encapsulation is also mentioned as a cause of mass rearing failures by decreasing the production of potential individuals to release [10]. Given the importance of assessing the potential for an endoparasitoid species as a biocontrol agent, knowledge on encapsulation and oviposition behaviour is crucial.

We examined in this paper some aspects of the parasitoid host immune relationship between *Pseudapanteles dignus* (Muesebeck) (Hymenoptera: Braconidae) and the tomato leafminer, *Tuta absoluta* (Meyrick) (Lepidoptera: Gelechiidae). *P*. *dignus* is a solitary endoparasitoid reported for few gelechiids, including *T*. *absoluta* [11, 12, 13], a tomato key pest native from South America, and since 2006, invasive in Eurasia and North Africa [14, 15, 16]. Laboratory and field studies done on *T*. *absoluta*—*P*. *dignus* interaction pointed out the potential of this parasitoid as a biocontrol agent against the pest in tomato crops [17, 18, 19].

The objectives of this study were: 1) to describe *P*. *dignus* early immature stages (egg and larva) encapsulation by *T*. *absoluta*, and 2) to determine the occurrence of self-superparasitism and the rate of escaping to encapsulation of this parasitoid. We addressed the hypothesis that self-superparasitism increases *P*. *dignus* successful development as a solitary endoparasitoid, by encapsulating totally or partially supernumerary immature individuals. We conclude that knowledge on encapsulation and superparasitism reported here may be useful to *P*. *dignus* rearing protocols for its mass production.

## Material and Methods

### Insect Colonies

*Tuta absoluta* and *P*. *dignus* colonies were established in the laboratory following Luna *et al*. [16] from damaged leaves collected in tomato crops located in La Plata, Buenos Aires, Argentina (34° 58’ S, 57° 59’ W). Specific permissions were neither required for collecting the material nor field studies injured endangered or protected species.

Leaves were kept in plastic boxes (500 ml) until *T*. *absoluta* pupation. Pupae were placed in meshed cages (30× 30× 30 cm), and when emerged, adults were fed with a honey solution (50%) *ad libitum*. Periodically, three-expanded leaves potted tomato plants were provided to adults for oviposition, and to start a new generation.

*P*. *dignus* colony was originated from cocoons (pupae) emerged from *T*. *absoluta* larvae collected in the field, and kept until wasp emergence in polystyrene Petri dishes (50 ml). Couples of adult wasps were placed in polypropylene cups (7.5 cm diameter and 18 cm height), provided with a honey solution (50%) as food, and batches of *T*. *absoluta* larvae settled in tomato leaves to oviposit. Exposed host larvae were maintained as described for healthy *T*. *absoluta* larvae in plastic boxes until *P*. *dignus* wasps were obtained, which were used in the experiments.

Both colonies were held in a walk-in environmental room at 25 ± 2°C, 70 ± 5% relative humidity and L14:D10 photoperiod, and checked every two days.

### Anatomical Aspects of Immature *P*. *dignus* Development and Detection of Encapsulation by *T*. *absoluta*

We conducted an experiment to study the sequence of events involved in the *P*. *dignus* encapsulation process by *T*. *absoluta*. The experimental unit consisted of a polypropylene cup (7.5 cm diameter and 18 cm height), where 10 *T*. *absoluta* larvae (second and third instar stages), installed in tomato leaf mines, were exposed to one *P*. *dignus* female (48-h old and 24-h mated), provided with 50% honey solution. Oviposition was allowed for 24 h to ensure parasitism, as previously reported [20]. At the next day, exposed hosts were transferred to polystyrene Petri dishes (10 cm diameter and 2 cm height) and kept alive until dissections. In total, 40 replicates were mounted, yielding a total of 40 wasp females and 400 *T*. *absoluta* larvae used. *T*. *absoluta* larvae were conditioned with fresh tomato foliage as food and maintained in an acclimatized walk-in rearing room as described above.

**Fig 1:**
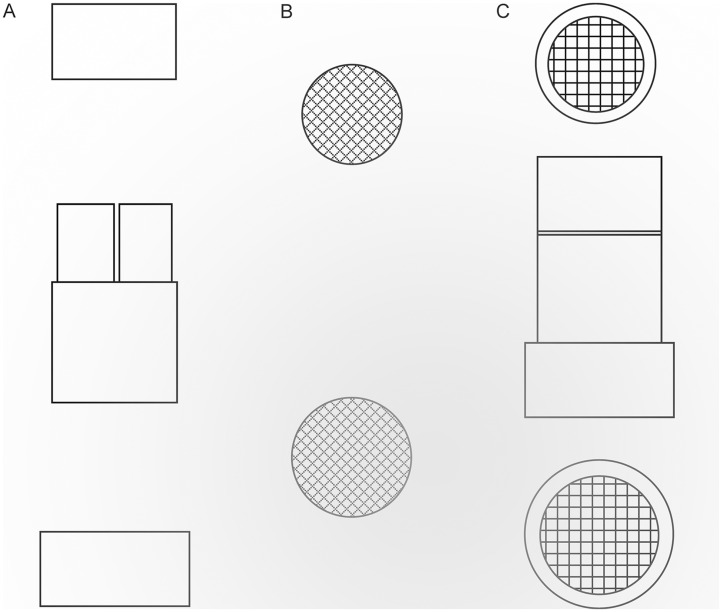
Design of the stainless steel capsule used to prepare *Pseudapanteles dignus* eggs and larvae for scanning electronic microscope (SEM) study. (**A**) Capsule in front view, opened. (**B**) Serigraphic- mesh pieces covering the internal sides of capsule openings in plan view. (**C**) Capsule in front view and plan view, closed.

Exposed hosts were dissected *in vivo* suspended in a drop of Ringer’s saline on a concave glass slide under a BX51 stereoscope microscope (Olympus UK Ltd.), from 0 to 5 d after *P*. *dignus* oviposition (intervals of 16, 24 to 36, 48 to 96 h), and daily up to 168 h, to search for parasitoid immature stages and detect encapsulation. Samples of immature parasitoids were prepared for observation using light (Nikon E200, Nikon Corporation) and scanning electron microscopy (SEM). For the SEM study, we followed the protocol described by Hayat [20]. Immediately after dissections, the parasitoids were individually transferred for fixation into a concave glass slide with a 2.5% glutaraldehyde solution in phosphate buffer (1M pH 7.5) and held for 4 h at 4°C. Afterward, since *P*. *dignus* immature stages are minute of size (<1 mm of length), the preparation for critical-point dry was carried out by relocating each parasitoid specimen individually in a 316 L- stainless steel vial designed for this study (11 mm diameter and 20 mm high) covered inside with a 100% polyester serigraphic mesh to avoid the loss of material (Fig 1).

Insects were dehydrated in 11 15-minutes changes in a graded acetone-distilled water series (2.5, 5, and then a range of 10 to 90%), and two hours in 100% acetone analytical grade. After dehydration and critical-point drying, the parasitoids were extracted from the vial with a fine brush and carefully mounted for gold coating using a microscope.

The encapsulation response by *T*. *absoluta* to *P dignus* was corroborated and described. Three degrees of *P*. *dignus* encapsulation were distinguished: encapsulated (totally covered by hemocytes and immobile), partially encapsulated (loose hemocytes aggregation attached to the parasitoid´s body that still remained its mobility), and non-encapsulated. The number of *P*. *dignus* individuals per host and the degree of encapsulation were recorded.

### Superparasitism and Egg/Larval Escape to Parasitism

From dissections, some aspects of the *P*. *dignus* reproduction behavior were analyzed: 1) superparasitism, as the proportion of host larvae containing one to the maximum number of immature *P*. *dignus* registered upon dissections, and 2) encapsulation rate, as the proportion of encapsulated larvae over the number of P. *dignus* larvae found per host. To analyze superparasitism, the number of immature *P*. *dignus* found upon dissections from 16 to 48 hours intervals were considered, since supernumerary *P*. *dignus* could be eliminated by host encapsulation and resorption after that period.

### Statistical Analyses

Means and standard errors were calculated for each variable. A one way-ANOVA was performed to compare the proportion of *T*. *absoluta* larvae (dependent variable) among the number of immature *P*. *dignus* registered per host (predictor variable). Previously, a test of homogeneity of variances was used (Levene´s test). Means were separated using a Student-Neuman-Keul (SNK) test with α = 0.05.

The rate of encapsulation of immature *P*. *dignus* by *T*. *absoluta* was analyzed by fitting a logistic regression between the proportion of parasitized hosts (*p*) and the number of parasitoid individuals per host (*x*). Because of the proportion of parasitism is a dependent categorical variable (two categories or codes, 0 = non encapsulated, 1 = encapsulated), which errors are assumed to be binomially distributed, the logistic model is more suitable [21]. The model is described by a function with two parameters, ß_0_ and ß_1_:

$$p\;=\;\frac{e^{\beta \text{0 +β1 x}}}{1+\;\;e^{\beta \text{0 +β1 x}}}$$

where the parameter ß_1_ is similar to a slope parameter (ß_1_ equal, minor o greater than zero indicates none, negative or positive relationship, respectively).

The program Statistica [22] was employed for all statistical analysis. Proportions were arcsin- transformed prior to all analyses.

## Results

### *P*. *dignus* Immature Development and Detection of Encapsulation by *T*. *absoluta*.

*In vivo* host dissections under the light microscope allowed observing the chronology of the immature *P*. *dignus* development when parasitizing *T*. *absoluta* larvae (Table 1).

**Table 1:** Development of immature *Pseudapanteles dignus* stages found upon host necropsies after 16, 24 to 48, 48 to 96, 96 to 120 and > 120 hours of *Tuta absoluta* larval parasitation.

Necropsies intervals (hours)	*Pseudapantales dignus* immature stage (%)
16	Egg (100)
24–36	Egg (1.6); early first larval instar (97.5); late first larval instar (0.9)
48–96	Early first larval instar (12.2); late first larval instar (85.4); second larval instar (2.4)
96–120	Early first larval instar (9.5); late first larval instar (52.4); second larval instar (28.6); third larval instar (9.5)
> 120	Mostly second and third larval instars (not measured)

The egg is deposited in the hemocele and the stage is nearly completed at 24 hours. The first larval instar was the longer lasting (ca. 96 hours). By the fifth day after oviposition, ca. 40% of larvae reached the second and third larval instars. Four out of 40 females failed ovipositing and then the hosts exposed were eliminated for further analyses.

**Fig 2:**
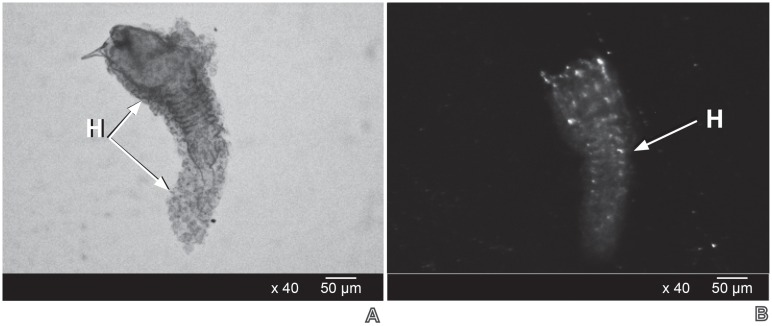
Light microscope (A) and light stereoscope microscope (B) appearance of the early first larval stadium of *Pseudapanteles dignus* encapsulated by hemocytes (H) of *Tuta absoluta*.

**Fig 3:**
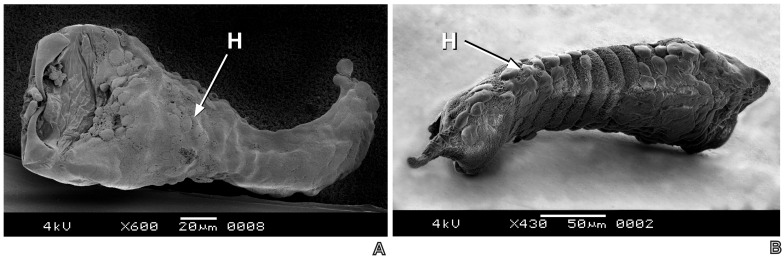
SEM images of the early first larval stadium of *Pseudapanteles dignus* showing (A) total, and (B) partial encapsulation by hemocytes aggregation of the host, *Tuta absoluta*.

**Fig 4:**
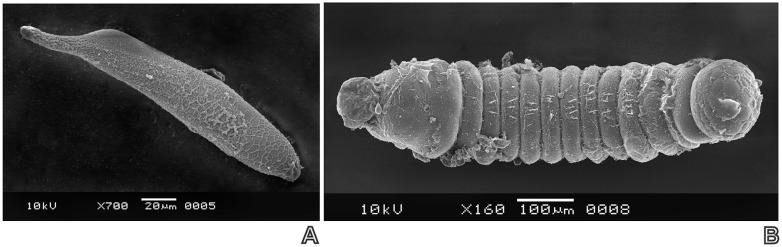
SEM images of non-encapsulated *Pseudapanteles dignus*. (**A**) egg and (**B**) late first larval stadium.

Necropsies evidenced that only the early *P*. *dignus* first larval instar (24–36 hour- old) had symptoms of encapsulation (n = 78 parasitized host larvae) (Figs 2 and 3), meanwhile neither the egg and nor late larval instars were encapsulated (Fig 4).

Hemocytes were adhered around the parasitoid body, with a flattened and symmetrically spread aspect, in accordance to as a granulocyte-type description. Light and SEM studies revealed the three degrees of encapsulation, from a dense capsule to partially encapsulated, leaving the parasitoid larva partly free and mobile, being still alive. The capsule did not show signs of melanization.

### Superparasitism and Escape to Encapsulation by the Host

**Fig 5:**
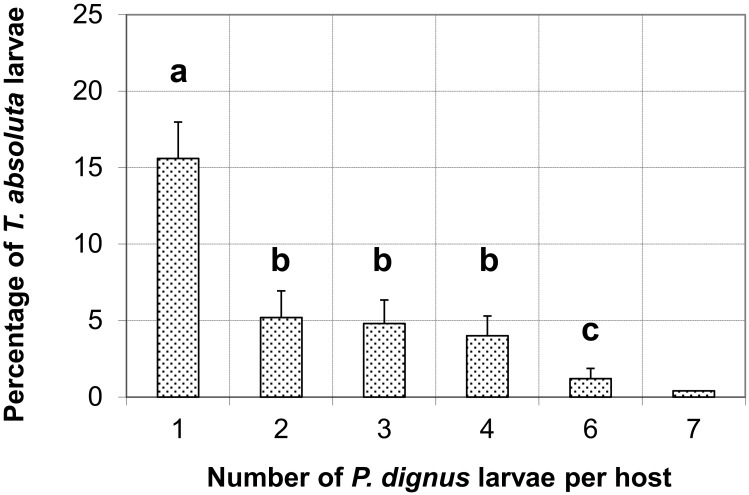
Mean percentages (+ SE) of *Tuta absoluta* larvae with one or more immature *Pseudapanteles dignus* after 16 to 48 hours of parasitation. Significant differences (*P* < 0.05) between means are indicated with different letters.

At 16 and 24 to 36 hour-intervals dissections, *P*. *dignus* predominantly behaved as a solitary species, but superparasitism also occurred. Approximately 50% of the parasitized *T*. *absoluta* larvae significantly had only one parasitoid egg deposited (n = 78, one-way ANOVA, *F* (5,114) = 11.57; *p*< 0.01). Supernumerary parasitization yielded mainly two to four *P*. *dignus* immatures per host, although a host with up to seven parasitoid eggs was occasionally registered (Fig 5 and S1 Table).

**Fig 6:**
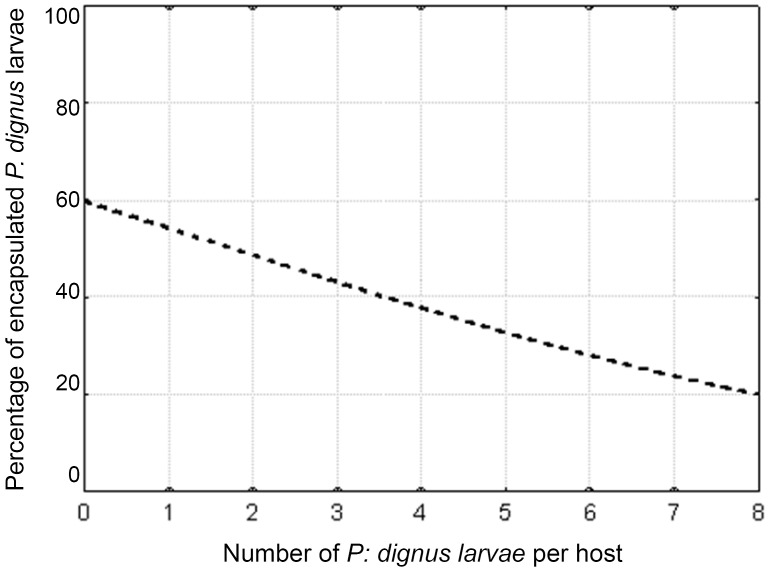
Percentage of encapsulated *Pseudapanteles dignus* early first larval stadium by *Tuta absoluta*, as a function of the number of the immature parasitoids per host larvae. Model: logistic regression (logit) y = exp (0.39 + (- 0.22) x)/(1+ exp (0.39 + (-0.22)x)).

A single parasitized *T*. *absoluta* larva could hold both encapsulated and non-encapsulated *P*. *dignus* larvae. Levels of encapsulation were significantly decreasing (Fig 6 and S2 Table) as the number of *P*. *dignus* larvae per host increased (Logistic regression (logit), ß_1_ = -0.22; Χ^2^: 124.34; *p*< 0.001).

## Discussion

The present work provides evidences of the anatomical and immunological aspects in the *P*. *dignus-T*. *absoluta* interaction. The first larval instar of *P*. *dignus* was found floating freely in the body cavity of the host, and the anatomical description was coincident with that reported by Cardona and Oatman [12] for other host, the tomato pinworm *Keiferia lycopersicella* (Walsingham) (Lepidoptera: Gelechiidae). The mature *P*. *dignus* first larval instar is reached ≈ 1 day longer in *T*. *absoluta* than in *K*. *lycopersicella*.

Only the early *P*. *dignus* first-larval instar is encapsulated by *T*. *absoluta*, meanwhile other instars (egg and older larval instars) escape from this immunological response. The capsule is formed by aggregated, granulocyte- or hyperphagocytic-type hemocytes described for other Lepidoptera species [2, 23], and did not melanize. The finding that the *P*. *dignus* egg stage is not encapsulated may result from its rapid development (< 24 h after oviposition). A fast egg parasitoid hatching is considered a strategy to anticipate the host immune system reaction, an event that usually takes longer time [1]. The presence of layers of proteins or virus-like particles adhered to the parasitoid egg’s surface has been pointed out also as preventing encapsulation in braconid wasp species [2, 6, 24, 25]. Although this was not explored in the present study, it would deserve further researching.

In solitary parasitoid species, only one individual larva is able to successfully complete its development in a host. If more than a single egg is laid in a host by itself or a conspecific female, the supernumerary larvae will be eliminated [5, 26]. In this study we found that the solitary endoparasitoid *P*. *dignus* superparasitizes *T*. *absoluta*, as it does in *K*. *lycopersicella* [12]. The decreasing rate of *P*. *dignus* larval encapsulation for single *vs*. multiple-parasitized *T*. *absoluta* larvae resulted more frequent than that observed for *K*. *lycopersicella* [12]. This braconid has been reported as a moderately synovigenic, time-limited species, with a mean daily oviposition rate of ≈ 8 female eggs per female [16, 17]. These reproductive traits allow females discarding eggs through self-parasitism to increase the likelihood of offspring to survive in parasitized hosts [27].

The maximum number of *P*. *dignus* immature parasitoids found for *T*. *absoluta* was greater than that reported for Cardona and Oatman [12], whom reported up to four immatures per host when reared on *K*. *lycopersicella*. The known mechanisms involved in reducing the brood size in solitary parasitoids are: 1) fighting and killing by siblings (usually during the first larval instar), and 2) superparasitism, that helps overcoming the host´s hemocytic reaction for the surviving larva [5]. Based on the *in vivo* dissection study done with the light microscope, evidences for fighting among parasitoid juveniles were not detected. Otherwise, many supernumerary larvae were observed as encapsulated. Thus, we conclude that multiple oviposition by *P*. *dignus* is an strategy to successfully parasitize *T*. *absoluta*.

Parasitoid-host physiological interactions help in determining host suitability and especially for endoparasitoids, the host specificity [1, 28]. Cui *et al*. [29] classified hosts as: permissive (allow the successful development of the parasitoid inside host), semi-permissive (partial, some can escape from host defenses), and non-permissive (not able to develop inside host). In this paper we showed that *T*. *absoluta* is a semi-permissive host for *P*. *dignus*. This braconid is considered an oligophagous species, since it has been reported parasitizing only members of gelechiid lepidopterans, including *T*. *absoluta*, *K*. *lycopersicella*, the potato tuberworm, *Phthorimaea operculella* (Zeller), and the pepper flower-bud moth, *Symmetrischema capsica* (Bradley and Polovny) [12, 13, 14]. Selection of hosts probably led to a narrow host range species with less developed defense reactions, what enhanced its survival chances.

In conclusion, our laboratory studies revealed that *P*. *dignus* early-first-instar larvae can be encapsulated by *T*. *absoluta* larvae, displaying this parasitoid a mechanism of superparasitism to limit the host capacity to encapsulate supernumerary parasitoid larvae per host. We also found that *T*. *absoluta* acts as a semi-permissive host for *P*. *dignus*. To increase knowledge of the use of *P*. *dignus* as a potential biocontrol candidate for *T*. *absoluta*, next research should address the outcome of the encapsulation and superparasitism under field conditions, since they can be affected by other abiotic and biotic factors, as temperature, host and parasitoid species densities, and by virus-like particles and other symbiontic infections in the parasitoid, among others.

## Supporting Information

S1 Table Summary of data for the number of immature *Pseudapanteles dignus* recorded in *Tuta absoluta* larvae after 16 to 48 hours of parasitation. (DOCX)

S2 Table Dataset of the proportion of encapsulated *Pseudapanteles dignus* early first larval stadium per *Tuta absoluta* larvae to perform the logistic regression showed in Fig 6. (DOCX)
