# Supplementary material for: Encapsulation and Self-Superparasitism of Pseudapanteles dignus (Muesebeck) (Hymenoptera: Braconidae), a Parasitoid of Tuta absoluta (Meyrick) (Lepidoptera: Gelechiidae)
Source: PLoS One. 2016 Oct 12;11(10):e0163196. doi: 10.1371/journal.pone.0163196 (PMC5061380; doi:10.1371/journal.pone.0163196)
Supplement: S1 Table — (DOCX) [file pone.0163196.s001.docx]

**Supporting Information S1**

**S1 Table. Summary of data for the number of immature *Pseudapanteles dignus* recorded in *Tuta absoluta* larvae after 16 to 48 hours of parasitation.**

(XLSX)
