# Supplementary material for: Encapsulation and Self-Superparasitism of Pseudapanteles dignus (Muesebeck) (Hymenoptera: Braconidae), a Parasitoid of Tuta absoluta (Meyrick) (Lepidoptera: Gelechiidae)
Source: PLoS One. 2016 Oct 12;11(10):e0163196. doi: 10.1371/journal.pone.0163196 (PMC5061380; doi:10.1371/journal.pone.0163196)
Supplement: S2 Table — (DOCX) [file pone.0163196.s002.docx]

**Supporting information file S2**

**S2 Table. Dataset of the proportion of encapsulated *Pseudapanteles dignus* early first larval stadium per *Tuta absoluta* larvae to perform the logistic regression showed in Fig 6.**

(XLSX)
